# Positive peritoneal swab in SARS-CoV-2 patients undergoing abdominal emergency surgery: effect or cause?

**DOI:** 10.1007/s15010-022-01785-z

**Published:** 2022-03-02

**Authors:** Dario Tartaglia, Andrea Barberis, Federico Coccolini, Mauro Pistello, Mariangela Rutigliani, Massimo Chiarugi

**Affiliations:** 1 General, Emergency and Trauma Surgery Department, Pisa University Hospital, Pisa, Italy; 2 Department of Abdominal Surgery, General and Hepatopancreatobiliary Surgery Unit, Galliera Hospital, Genoa, Italy; 3 Virology Unit, Pisa University Hospital, Pisa, Italy; 4 Department of Laboratory and Service, Histological and Anatomical Pathology, E.O. Galliera Hospital, Genoa, Italy

**Keywords:** Coronavirus, COVID-19, Laparoscopy, Laparotomy, SARS-CoV-2, Peritoneal fluid

## Abstract

**Purpose:**

The presence of the SARS-CoV-2 in the peritoneal fluid is a matter of debate in the COVID-19 literature. The study aimed to report the prevalence of SARS-CoV-2 in the peritoneal fluid of patients with nasopharyngeal swab tested positive for SARS-CoV-2 undergoing emergency surgery and review the literature.

**Methods:**

The present study was conducted between March 2020 and June 2021. Diagnosis of SARS-CoV-2 positivity was confirmed by preoperative real-time reverse transcriptase-polymerase chain reaction (RT-PCR).

**Results:**

Eighteen patients with positive nasopharyngeal swabs were operated in emergency in two third-level Italian hospitals. In 13 of these patients (72%), a peritoneal swab was analyzed: SARS-CoV-2 RNA was found in the abdominal fluid of two patients (15%). Neither of them had visceral perforation and one patient died. In ten patients with negative peritoneal swabs, visceral perforation and mortality rates were 30% and 20%, respectively.

**Conclusion:**

SARS-CoV-2 peritoneal positivity is rare. Abdominal surgery can, therefore, be safely performed in patients with COVID-19 using standard precautions. The correlation with a visceral perforation is not evaluable. The clinical outcomes seem uninfluenced by the viral colonization of the peritoneum. Assessment in large series to provide definitive answers about the involvement of the SARS-CoV-2 in the peritoneum will be challenging to coordinate.

## Background

With almost 6,000,000 deaths and more than 400,000,000 confirmed cases worldwide, the global pandemic of COVID-19, caused by severe acute respiratory syndrome coronavirus 2 (SARS-CoV-2), has irrevocably changed the social and health direction of humankind. COVID-19 presents a wide spectrum of symptoms, from severe respiratory distress caused by interstitial pneumonia to significant gastrointestinal complications. It has been demonstrated that SARS-CoV-2 can employ the angiotensin-converting enzyme 2 (ACE2) to invade host cells as a cell surface receptor, and thus invade different tissues in the body. Therefore, tissues with greater expression of ACE2 are a potential target for the virus. The presence of SARS-CoV-2 in the peritoneal fluid is a matter of debate in recent COVID-19 literature. It has been speculated that, if SARS-CoV-2 infects the abdominal cavity, there may be several implications: aerosolization of the viral particles following electrocauterization or pneumoperitoneum evacuation during laparoscopy; a worse outcome for COVID-19 patients undergoing emergency surgery; and a possible increased risk of intestinal ischemia. The present evidence is inconclusive, as many contrasting results have been shown. Furthermore, only case reports and small series have been reported in the literature. Thus, we attempted to report the prevalence of SARS-CoV-2 in the peritoneal fluid in a series of patients with a SARS-CoV-2-positive nasopharyngeal swab undergoing emergency surgery in two Italian third-level hospitals. We also compared our results with data taken from the literature.

## Methods

To identify the presence of SARS-CoV-2 in the peritoneal fluid, we obtained several swabs during emergency abdominal surgery in patients with SARS-CoV-2 isolated in the nasopharyngeal swab between March 2020 and June 2021. Nasopharyngeal samples were collected using Copan FLOQSwabs® and a sterile tube containing Copan’s Universal Transport Medium (COPAN Diagnostics Inc., Murrieta, CA, USA). Viral positivity was defined in the case of real-time reverse transcriptase-polymerase chain (RT-PCR) detection of viral RNA. Peritoneal and rectal swabs consisted of FLOQSwabs® with molded breaking point screw-cap tubes filled with 1 ml of liquid Amies medium (eSwab®, COPAN Diagnostics Inc., Murrieta, CA, USA). Two samples were obtained at the outset of the operations, by soaking swabs’ tip in the peritoneal fluid. In the case of laparoscopy, swabs were introduced through a trocar. The real-time RT-PCR used was a CE-IVD (in vitro diagnostic)-labeled system marketed by Arrow/Seegene, targeting genes E (envelope glycoprotein), N (nucleocapsid) and RdRp (RNA polymerase) of SARS-CoV-2 RNA genome, and detecting up to 100 SARS-CoV-2 RNA genome copies/reaction. Nucleic acid extraction of nasopharyngeal and abdominal swabs was performed with a universal extraction kit, produced by the same manufacturer, and validated on a wide array of biological materials. The paper has been worded in line with the STROBE Statement. All procedures performed were in accordance with the ethical standards of the institutional and/or national research committee and with the 1964 Helsinki Declaration and its later amendments or comparable ethical standards. The study was approved by the institutional board. Demographic, clinical and outcome parameters were collected and described. After revision of the literature, two groups were identified: patients with negative peritoneal swabs and those with positive ones. Groups were compared using *t* test or Mann–Whitney *U* test, where appropriate, for numerical variables and Fisher's exact test for categorical variables. Differences were defined as statistically significant when the *p* value was < 0.05. XLstat was used for statistical analysis.

## Results

Overall, 18 COVID-19 patients out of 1807 underwent emergency surgery during the considered study period (1%). The median age was 71 (IQR: 18–95) years (Table 1). Male patients accounted for 12 of the 18 (67%) cases. Co-morbidities were present in 78% of patients (Table 1). A thoracic CT scan was performed in 15 of the 18 cases (83%) and identified COVID-19 interstitial pneumonia in 11 cases (73%). The median time between nasopharyngeal swab positivity and surgery was 1.5 days (IQR: 0–76). Reasons and types of surgery are listed in Table 2. The peritoneal swab was taken in 13 of the 18 patients (72%). The median time between nasopharyngeal swab positivity and the peritoneal swab was 2 days (IQR: 0–57 days). In 2 cases (17%), SARS-CoV-2 was isolated in the peritoneal fluid. The CT obtained with the nasal swabs were around 30 for the three genes and those obtained with the abdominal swabs were five to six times higher, indicating that the virus present in the abdomen was about 100 times lower than the upper respiratory tract. The pathologies leading to surgery in those two patients were ischemic colitis and adhesive small bowel occlusion, respectively. A rectal swab was available in 4/18 cases: all of these were negative for SARS-CoV-2 and were not associated with peritoneal positivity. None of the patients with positive peritoneal swabs had visceral perforation and one, the patient with ischemic colitis, died due to cardio-pulmonary failure. In the other 11 patients, three had intestinal perforation and two died (Tables 1, 2). In the overall cohort of cases, the postoperative course was complicated in 8 cases (44%): 6 (33%) presented major complications. Five out of 18 patients (28%) died due to multiorgan failure for ongoing sepsis related to abdominal processes (*n* = 4) and respiratory failure (*n* = 1). The median postoperative hospital stay was 15 days (IQR: 3–106 days). In terms of postoperative course, the comparison between nasopharyngeal SARS-CoV-2-positive patients (*N* = 18) and age-matched nasopharyngeal SARS-CoV-2-negative subjects (*N* = 77) undergoing abdominal emergency surgery, showed a significantly different morbidity (44% vs 17%; *p* = 0.02), major complication rate (33% vs 5%; *p* = 0.001), mortality (28% vs 4%; *p* = 0.006) and postoperative hospital stay (mean ± standard deviation) (25.8 ± 29.5 vs 14 ± 39.6 days; *p* = 0.001).

**Table 1:** Demographic and clinical characteristics of patients with COVID-19 patients undergoing emergency surgery

	COVID-19 patients undergoing emergency surgery OVERALL, *N* = 18	COVID-19 patients undergoing emergency surgery WITH peritoneal swab, *N* = 13	COVID-19 patients undergoing emergency surgery WITHOUT peritoneal swab, *N* = 5
Median age (IQR) years	71 (18–95)	75 (18–95)	71 (67–73)
Male gender (*N*, %)	12 (67)	7 (54)	5 (100)
Comorbidity (*N*, %)	14 (78)	10 (77)	4 (80)
Metabolic disorders	8 (44)	5 (38)	3 (60)
Cardiopathy	6 (33)	5 (38)	1 (20)
Kidney disease	4 (22)	4 (31)	0
Pneumopathy	4 (22)	3 (23)	1 (20)
Vasculopathy	3 (17)	3 (23)	0
Rheumatic disorders	1 (6)	1 (8)	0
Thoracic CT scan (*N*, %)	15 (83)	12 (92)	3 (60)
COVID-19 pneumonia (*N*, %)	11 (73)	9 (69)	2 (40)
Reasons of surgery (*N*, %)			
Ischemic colitis	3 (17)	3 (23)	0
Acute appendicitis	3 (17)	2 (15)	1 (20)
Ischemic intestinal perforation	2 (11)	2 (15)	0
Complicated sigmoid diverticulitis	2 (11)	1 (8)	1 (20)
Obstructing colonic tumor	2 (11)	1 (8)	1 (20)
Adhesive small bowel occlusion	1 (11)	1 (8)	0
Biliary ileus	1 (11)	1 (8)	0
Incarcerated inguinal hernia	1 (11)	1 (8)	0
Spontaneous hemoperitoneum	1 (11)	1 (8)	0
Jejunal diverticular perforation	1 (11)	0	1 (20)
Duodenal bleeding ulcer	1 (11)	0	1 (20)

*IQR* interquartile range

**Table 2:** Surgical and postoperative findings of COVID-19 patients undergoing emergency surgery

	COVID-19 patients undergoing emergency surgery OVERALL, *N* = 18	COVID-19 patients undergoing emergency surgery WITH peritoneal swab, *N* = 13	COVID-19 patients undergoing emergency surgery WITHOUT peritoneal swab, *N* = 5
Type of surgery (*N*, %)			
Colic resections	7 (39)	5 (38)	2 (40)
Appendectomy	3 (17)	2 (15)	1 (20)
Small bowel resection	2 (11)	1 (8)	1 (20)
Loop ileostomy	1 (6)	1 (8)	0
Enterotomy	1 (6)	1 (8)	0
Lysis of peritoneal adherences	1 (6)	1 (8)	0
Packing with open abdomen	1 (6)	1 (8)	0
Hernia repair	1 (6)	1 (8)	0
Ulcer repair with suture	1 (6)	0	1 (20)
Median IIME between nasopharyngeal swab positivity and surgery (IQR) (days)	2 (0–57)	2 (0–57)	1 (0–46)
Peritoneal positivity (*N*, %)	–	**2 (15)**	–
Morbidity (*N*, %)	8 (44)	6 (46)	2 (40)
Major complications (*N*, %) (Clavien and Dindo classification > 2)	6 (33)	4 (31)	2 (40)
Mortality (*N*, %)	5 (28)	3 (23)	2 (40)
Postoperative hospital (IQR) (days)	15 (3–106)	14 (3–40)	18 (5–106)

Bold means significantly different (*p* < 0.05)

*IQR* interquartile range

From the literature, 50 patients with positive nasopharyngeal swabs for SARS-CoV-2 undergoing surgery had their peritoneal swabs analyzed: 4 (8%) were positive. Demographic and clinical comparisons are reported in Table 3. There were significant differences between patients with peritoneal negative swabs and positive ones in terms of visceral perforations (0 vs 50%; *p* = 0.004) and morbidity (0 vs 50%; *p* = 0.004).

**Table 3:** Demographic and clinical characteristics of patients with nasopharyngeal swab positive for SARS-CoV-2 undergoing abdominal surgery from the literature

	PERITONAL SWAB *NEGATIVE* FOR SARS-CoV-2, *N* = 46 (92%)	PERITONEAL SWAB *POSITIVE *FOR SARS-CoV-2, *N* = 4 (8%)	*p* value
Mean age (SD) years	34* (12.1)	43 (36.2)	0.24
Female gender (*N*, %)	37 (80%)	2 (50%)	0.18
Comorbidity (*N*, %)	28** (70%)	2 (50%)	0.58
COVID-19 pneumonia (*N*, %)	13 (28%)	2 (50%)	0.57
Visceral perforation (*N*, %)	0	2 (50%)	**0.004**
Laparoscopy (*N*, %)	2 (4%)	2 (50%)	**0.02**
Morbidity (*N*, %)	0	2 (50%)	**0.004**
Mortality (*N*, %)	0	1 (25%)	0.08

Bold means significantly different (*p* < 0.05)

*SD* standard deviation

*1 missing

**6 missing

## Discussions and conclusion

The presence of SARS-Cov-2 in the peritoneal fluid is a lively matter of debate in the recent COVID literature. Several case studies have not detected SARS-CoV-2 in the peritoneal fluid [1–7]. In a large series consisting of 34 pregnant women with positive SARS-CoV-2 nasopharyngeal swab undergoing cesarean section, Jakimiuk et al. reported that all peritoneal fluid samples tested negative for SARS-CoV-2 ribonucleic acid [5]. Similarly, El Shamy et al. did not find RNA viral particles from the peritoneal effluent of 10 COVID-19 patients with end-stage kidney disease experiencing acute peritoneal dialysis [2]. Other small case series and case reports consisting of 12 patients produced similar findings [1, 4, 6, 7].

Other authors reported the isolation of the virus in the peritoneum, suggesting the possibility that the virus can spread through the serosa membranes [8–12]. Overall, six patients were found positive for SARS-CoV-2 in the peritoneum: two of them did not undergo abdominal surgery [10, 11]. The literature shows that the prevalence of SARS-CoV-2 in the peritoneal fluid is approximately 8%, which is lower than that of our results: 15%.

A recent review by Cheruiyot et al. reported 19 studies (15 case reports and 4 case series) comprising 29 COVID-19 patients. They found that 11 patients (38%) were positive for viral RNA in different abdominal fluids and tissues: peritoneal fluid, bile, ascitic fluid, peritoneal dialysate, duodenal wall, and appendix. Thus, the authors concluded that larger studies were required; no evidence that supports the possibility that SARS-CoV-2 can be aerosolized and transmitted to other individuals is available due to conflicting results [13]. The potential role of the gut in COVID-19 infection has been amply demonstrated with a stool virionic RNA positivity ranging between 20 and 47% of cases. Surprisingly, SARS-CoV-2 fecal shedding seems to be prolonged, persisting up to several weeks after respiratory swab negativization. Barone et al. identified that a visceral ischemic insult was associated with a threefold increased relative risk of peritoneal fluid positivity, albeit in the absence of a relevant statistical correlation (RR 3.00 [95% CI 0.39–23.07; *p* = 0.29]). Furthermore, according to their available literature, 50% of patients with preoperative findings of ischemia and/or indirect signs of microvascular injury (ulceration, bleeding) had positive peritoneal swabs. In contrast, only 16% of patients without preoperative signs of ischemia showed RT-PCR SARS-CoV-2 abdominal positivity [14].

Among the 50 patients reported in the literature, we identified a significantly higher rate of visceral perforations and morbidity in COVID-19 patients with positive peritoneal swabs (*p* = 0.004) (Table 1). Increased morbidity may be, however, influenced by the high rate of COVID-19 pneumonia in the cohort of patients with peritoneal viral positivity. Although we could speculate that an intestinal discontinuity may be related to an increased odd of viral propagation into the peritoneal cavity, the two patients from our cohort presenting SARS-CoV-2 in the peritoneum did not show any visceral perforation.

Due to the increased risk of potential SARS-CoV-2 contaminated aerosol during laparoscopy or open surgery positivity, operating teams should take precautions during an operation on a positive patient, such as the use of full personal protective equipment, minimizing the number of medical personnel, evacuation of smoke with suction devices, and, in the case of laparoscopy, avoiding two‐way pneumoperitoneum insufflators to maintain pneumoperitoneum pressure and ventilation at the lowest possible levels. Based on the findings of our study, it is our opinion that patients with a positive peritoneal swab are unlikely to contaminate an operating room and that the adopted precautions are effective in protecting the exposed health workers from infection.

This study is limited by the small cohort of patients. The presence of viremia and the extent of viral load at the time of sampling in our patients were unknown. Furthermore, the accuracy of molecular tests for the measurement of viral RNA in tissue samples has not been characterized, and viral cultures were not performed. However, the present study is the first attempt to perform a multicenter study on this topic. We attempted to include as many centers as possible, but received very few answers, likely due to the organizational difficulties involved in collecting and analyzing peritoneal swabs from a significant cohort of patients during the pandemic crisis.

In conclusion, SARS-CoV-2 peritoneal positivity is rare. The correlation with visceral perforation is not evaluable. However, patients’ outcome does not appear to be influenced by viral colonization of the peritoneum. Surgery can be safely performed in patients with COVID-19 through standard precautions. An extensive series could have given more significant results, but a multicenter study will likely be challenging to organize.

## References

[1] Seeliger B, Philouze G, Benotmane I (2020). Is the severe acute respiratory syndrome coronavirus 2 (SARS-CoV-2) present intraperitoneally in patients with coronavirus disease 2019 (COVID-19) infection undergoing emergency operations?. Surgery.

[2] El Shamy O, Vassalotti JA, Sharma S (2020). Coronavirus disease 2019 (COVID-19) hospitalized patients with acute kidney injury treated with acute peritoneal dialysis do not have infectious peritoneal dialysis effluent. Kidney Int.

[3] Safari S, Keyvani H, Malekpour Alamdari N (2020). Abdominal Surgery in Patients With COVID-19: Detection of SARS-CoV-2 in Abdominal and Adipose Tissues. Ann Surg.

[4] Flemming S, Hankir M, Hering I (2020). Abdominal fluid samples (negative for SARS-CoV-2) from a critically unwell patient with respiratory COVID-19. Br J Surg.

[5] Jakimiuk AJ, Januszewski M, Santor-Zaczynska M et al. Absence of SARS-CoV-2 RNA in peritoneal fluid during surgery in pregnant women who are COVID-19 positive. J Minim Invasive Gynecol. 2021; 15:S1553-4650(21)00285-5. 10.1016/j.jmig.2021.06.006 PMC8205274 34144207

[6] Romero-Velez G, Pereira X, Zenilman A, Camacho D (2020). SARS-Cov-2 was not found in the peritoneal fluid of an asymptomatic patient undergoing laparoscopic appendectomy. Surg Laparosc Endosc Percutan Tech.

[7] AlAradi J, AlHarmi RAR, AlKooheji M (2021). SARS-CoV-2 in peritoneal swabs from asymptomatic patients undergoing emergency abdominal surgery. J Surg Case Rep.

[8] Barberis A, Rutigliani M, Belli F, et al. SARS-Cov-2 in peritoneal fluid: an important finding in the Covid-19 pandemic. *Br J Surg.* 2020; 107(10):e376. 10.1002/bjs.11816 PMC7405209 32687638

[9] Coccolini F, Tartaglia D, Puglisi A (2020). SARS-CoV-2 is present in peritoneal fluid in COVID-19 patients. Ann Surg.

[10] Passarelli VC, Perosa AH, De Souza Luna LK (2020). Detected SARS-CoV-2 in ascitic fluid followed by cryptococcemia: a case report. SN Compr Clin Med.

[11] Vischini G, D'Alonzo S, Grandaliano G, D'Ascenzo FM (2020). SARS-CoV-2 in the peritoneal waste in a patient treated with peritoneal dialysis. Kidney Int.

[12] Pani E, Collini L, Naselli A (2021). SARS-Cov-2 in peritoneal fluid of two children with COVID-19: a rare finding. J Paediatr Child Health.

[13] Cheruiyot I, Sehmi P, Ngure B (2021). Laparoscopic surgery during the COVID-19 pandemic: detection of SARS-COV-2 in abdominal tissues, fluids, and surgical smoke. Langenbecks Arch Surg.

[14] Barone M, Ippoliti M, Mucilli F (2021). SARS-CoV-2 peritoneal positivity and emergency surgery: is there any putative predisposing factor?. Updates Surg.

